# Conversion from cardiac resynchronization therapy to left bundle branch area pacing in a child with congenital heart disease and pacing-induced cardiomyopathy: A case report

**DOI:** 10.1016/j.hrcr.2026.04.006

**Published:** 2026-04-09

**Authors:** Wisam Abozaid, Shubhayan Sanatani, Sonia Franciosi, Sakethram Saravu Vijayashankar

**Affiliations:** Department of Pediatrics, The University of British Columbia, Vancouver, British Columbia, Canada, and Children’s Heart Center, British Columbia Children’s Hospital, Vancouver, British Columbia, Canada

**Keywords:** Left bundle branch area pacing, Conduction system pacing, Cardiac resynchronization therapy, Pacing-induced cardiomyopathy, Ventricular dyssynchrony, Pediatrics, Congenital heart disease, Complete AV block

**Key Teaching Points**

• Transvenous left bundle branch area pacing can be successfully performed in pediatric patients with complex anatomy and septal prosthetic material when cardiac resynchronization therapy–pacemaker fails.
• Left bundle branch area pacing can provide ventricular synchrony and functional recovery similar to cardiac resynchronization therapy–pacemaker implantation, as shown by normalized systolic function at 6 months.
• Even after long-standing atrioventricular block, viable conduction fibers may persist and be captured for physiologic pacing.

## Introduction

Conduction system pacing (CSP), including left bundle branch area pacing (LBBAP), is recognized as an alternative to conventional right ventricular (RV) pacing, aiming to mitigate pacing-induced cardiomyopathy (PICM) by minimizing ventricular dyssynchrony.^1^ We present a pediatric case of congenital heart disease (CHD) with PICM, previously managed with cardiac resynchronization therapy (CRT), which was successfully converted to LBBAP. After the procedure, the patient demonstrated a significant improvement in electrocardiographic and echocardiographic indices of ventricular function. This case highlights the feasibility of LBBAP in pediatric patients and its potential advantages over CRT in managing PICM.

## Case report

A 12-year-old, 38-kg boy with D-transposition of the great arteries and a muscular-to-inlet ventricular septal defect (VSD), surgically corrected neonatally with an arterial switch operation and VSD closure, developed postoperative complete atrioventricular (AV) block with a junctional escape rhythm. This necessitated implantation of an epicardial dual-chamber pacemaker. Medtronic unipolar epicardial leads were placed on the lateral right atrium and the anterosuperior right ventricle, each demonstrating appropriate acute thresholds. The leads were connected to a Adapta generator (Medtronic Inc., Minneapolis, MN). Within 1 week, he demonstrated clinically significant atrial undersensing, prompting surgical revision. The epicardial atrial lead was repositioned from the high right atrium to the lower right atrial free wall, resulting in improved P-wave amplitudes (2.0–2.8 mV) and excellent pacing thresholds. The pacemaker was programmed DDD at 100–210 beats/min, with stable device parameters on subsequent interrogations. He remained clinically asymptomatic (Ross Class I) for 3 months. Despite preserved functional status, he developed PICM characterized by progressive left ventricular (LV) systolic dysfunction and pronounced interventricular dyssynchrony. His ejection fraction (EF) declined from normal to 34%, accompanied by paradoxical septal motion and an abnormal global longitudinal strain pattern. Guideline-directed medical therapy (furosemide, spironolactone, enalapril, and carvedilol) was initiated. Remarkably, he remained physically active with age-appropriate exercise tolerance.

Over 4 years, he developed worsening ventricular function, with the EF reaching a nadir of 13% and severe LV dilation (LV end-diastolic dimension 5.1 cm; Z-score +7.5). He developed exertional intolerance consistent with New York Heart Association (NYHA) functional class II, and B-type natriuretic peptide (BNP) rose to 7351 pg/mL. These findings prompted an upgrade to epicardial biventricular pacing at age 5. A bipolar epicardial LV lead was positioned on the apical LV epicardium, and generator replacement was performed concurrently. The device was programmed DDD at 70–210 beats/min with synchronous biventricular activation, achieving the narrowest QRS morphology and optimal hemodynamic profile on echocardiography. After resynchronization, he demonstrated marked clinical and biochemical improvement: NYHA functional class I status, EF improving to 33%, and normalization of BNP values (15–126 pg/mL). Heart failure medications were subsequently optimized. Although PICM improved after CRT device implantation, LV systolic function remained partially impaired. During the year preceding his current presentation, despite relatively stable ventricular function (EF 33%–36%), he developed progressive exertional intolerance and reduced endurance consistent with NYHA functional class II status. This functional decline correlated with a rise in BNP to 234 pg/mL. His heart failure regimen was optimized, including carvedilol, sacubitril/valsartan, and spironolactone.

**Figure 1:**
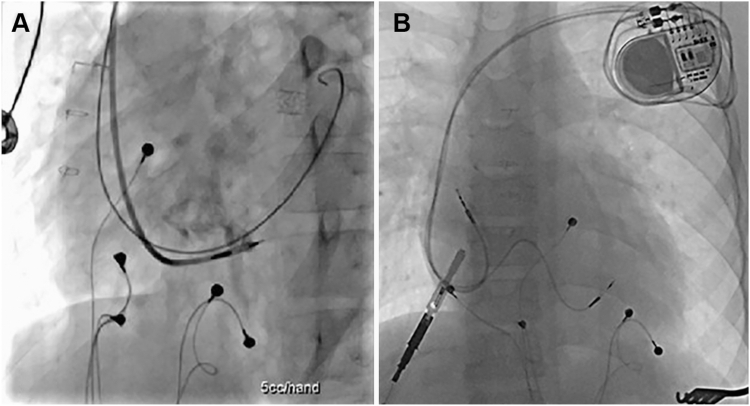
**A:** Septogram showing an optimal lead depth in the interventricular septum. **B:** Dual-chamber pacemaker with additional lead slack to accommodate anticipated teenage growth. The left bundle branch area pacing lead is positioned inferoapically, necessitated by the presence of a large nonconductive ventricular septal defect patch.

**Figure 2:**
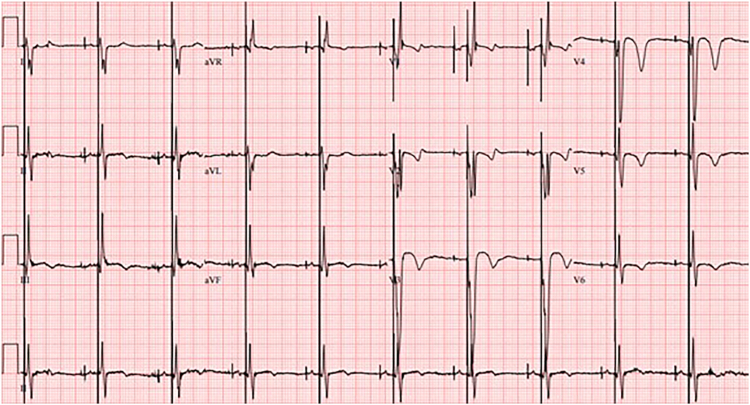
12-lead electrocardiogram postintervention showing atrial-paced, ventricular-paced rhythm at 60 beats/min with reliable capture of both atrial and ventricular leads. The tracing demonstrates a qR pattern in lead V_1_, late R-wave transition, discordance between leads aVL and aVR, and a QRS duration of 150 ms.

At age 12, he again presented with recurrent syncope. Device interrogation demonstrated RV lead failure characterized by inappropriate sensing, elevated impedance (>3000 Ω), and loss of ventricular capture; findings consistent with RV epicardial lead fracture and LV epicardial lead oversensing. Intermittent LV lead oversensing further compromised effective pacing despite progressive reduction in ventricular sensitivity (adjusted from 5.0 to 11.3 mV). For safety, the device was reprogrammed to asynchronous DOO mode at 80 beats/min, restoring reliable biventricular capture. Chest radiography revealed a new fracture in an atrial pacing lead (previously configured in unipolar mode), with no radiographic evidence of ventricular lead fracture. The patient was admitted to the cardiac intensive care unit for continuous telemetry monitoring and stabilization while maintained on his regular medications. Management options were discussed, including implantation of a transvenous CRT-pacemaker (CRT-P) vs a transvenous LBBAP system. A transvenous LBBAP system was selected as the primary physiologic pacing strategy, and the patient subsequently underwent transvenous pacing system revision the following morning. No His-Purkinje activity was detected, even when the pacemaker was turned off intermittently for short periods of time (∼5 seconds), precluding mapping of His bundle signals. The sheath was then positioned near the AV node using standard fluoroscopic landmarks, and initial attempts at LBBAP were performed at the standard anatomical location—approximately 1 cm apical to the presumed AV node. However, a large non-paceable area was encountered at this site, corresponding to the VSD patch placed during the patient’s neonatal surgery. The VSD patch margins were delineated electrically, and a more apical and posterior septal site was identified with acceptable initial pacing characteristics (discordance between leads aVR and aVL and W-pattern in lead V_1_). A Medtronic-3830 pacing lead was advanced and fixed at this location. An initial rise, followed by stable impedance, was observed at the final position. Electrocardiographic features included a qRʹ morphology in lead V_1_, a QRS duration of 130 ms (compared with 176 ms on his presentation), and a stimulus-to-LV activation time of 60 ms. Lead vector analysis demonstrated discordance between leads aVR and aVL, with R-wave transition occurring in leads V_4_–V_5_, likely reflecting inferoposterior septal positioning of the lead. A contrast septogram confirmed appropriate intraseptal deployment of the helix (Figure 1A and 1B), and fixation was deemed stable. Subsequently, a Medtronic active-fixation atrial lead was implanted in the right atrial appendage with satisfactory positioning and electrical parameters. An Azure DR dual-chamber generator (Medtronic Inc.) was connected to the newly implanted leads and programmed in DDD mode at 60–180 beats/min. The prior epicardial device was reprogrammed to ODO mode to prevent competitive pacing. The procedure was uncomplicated, and the patient was transferred to recovery in stable condition. He remained hemodynamically stable and was discharged on postoperative day 1. At 6-month follow-up, the patient remained asymptomatic, with stable device parameters and unchanged electrocardiographic findings from the immediate postimplantation period (Figure 2). Transthoracic echocardiography demonstrated preserved systolic function with an EF of 54%, only mild mechanical dyssynchrony at the LV apex, and global strain −14.7%, indicating improved ventricular performance under CSP. N-terminal pro-BNP levels were within normal limits, 124 pg/mL.

## Discussion

CSP, including His bundle pacing (HBP) and LBBAP, has emerged as a favorable strategy for permanent pacing in both adult and pediatric populations.^1^^,^^2^ CSP offers an alternative to conventional RV apical pacing, in patients with or without structural CHD, by closely replicating native ventricular activation and mitigating pacing-induced dyssynchrony.^2^ Single-site RV pacing has long been associated with an increased risk of PICM and progressive systolic dysfunction. Although the mechanism remains unclear, the leading hypothesis attributes PICM to ventricular dyssynchrony.^3^ Asynchronous ventricular activation leads to inefficient myocardial work, abnormal myofibrillar strain, maladaptive remodeling, and decline in contractile function. These effects, including mechanical dyssynchrony, activation of apoptotic pathways, and alterations in calcium handling with chronic RV pacing, have been documented previously.^4^ Prolonged paced QRS duration, irrespective of the pacing site, is also linked to the development of PICM, underscoring the role of electrical dyssynchrony in PICM pathogenesis.^5^^,^^6^ These limitations of conventional pacing strategies have driven interest in CSP to preserve physiologic ventricular activation and mitigate the adverse effects of electrical dyssynchrony. Although CSP has demonstrated superiority in minimizing both inter- and intraventricular dyssynchrony,^7^ its widespread adoption, particularly with HBP, remains limited because of challenges such as the technical complexity of achieving reliable His bundle capture, which entails a steep learning curve, as well as concerns regarding long-term lead performance, including elevated capture thresholds and a higher risk of lead instability or failure over time.^2^^,^^8^

LBBAP has gained broader acceptance as a preferred form of CSP, because of its technical ease compared with HBP, as well as its favorable electrical and procedural characteristics such as a large target area, technical simplicity, high success rates, preserved LV synchrony, fewer leads, and stable lead parameters.^1^^,^^9^ Since its initial description, LBBAP has increasingly been adopted as a practical and effective alternative to conventional biventricular CRT in the management of PICM.^10^ However, its application in the pediatric population remains limited by factors such as smaller body size, vascular access constraints, and the complexity of congenital cardiac anatomy. Despite these challenges, CSP is progressively supplanting biventricular CRT as the preferred strategy for both prevention and treatment of PICM, particularly in select pediatric patients where anatomical feasibility permits.^2^^,^^11^

Current evidence supporting LBBAP in the pediatric population is limited to case reports and small case series. In a study of 12 pediatric patients, of whom 11 had complete AV block and 1 had PICM, the latter patient demonstrated improved ventricular function after LBBAP. Across the entire cohort, postprocedural echocardiographic evaluation revealed improvement in LV end-diastolic dimension and Z scores, suggesting favorable effects on ventricular remodeling and chamber size.^12^ In a retrospective study of pediatric patients comparing LBBAP with RV septal pacing, LBBAP was associated with significantly narrower paced QRS durations and superior postoperative electrical and mechanical synchrony underscoring the potential of LBBAP to provide more physiologic ventricular activation in children requiring permanent pacing.^13^ In a study which included adult patients with CRT nonresponse, the LBBP group showed significant LV EF improvement and QRS duration shortening.^14^ Similar promising outcomes were reported in pediatric patients with PICM, all of whom had baseline high-degree AV block and underwent upgrade from conventional pacing systems to LBBAP. At a mean follow-up of 6 months, 5 of 6 patients demonstrated improvement in LV EF, whereas 1 patient remained a nonresponder to CSP.^15^ In a nonrandomized observational study, LBBAP was both feasible and safe in patients with CHD, with outcomes comparable to those in patients without CHD.^16^ Moore et al^7^ provide important evidence supporting the feasibility of CSP, including LBBAP, primarily in a population of adult patients with CHD, with limited pediatric representation. Our case expands on these findings by describing a child with CHD who experienced late failure of a previously effective epicardial CRT system, followed by successful transition to LBBAP as a rescue strategy. Unlike prior reports, this case demonstrates the feasibility of CSP in the setting of postsurgical CHD, including the presence of septal prosthetic material, and highlights unique pediatric considerations related to long-term device durability, growth, and evolving anatomy when conventional CRT is no longer viable.

To our knowledge, no previous pediatric reports describe conversion from a previously successful epicardial CRT system that subsequently failed to an LBBAP system for ongoing PICM management. At 6-month follow-up, our patient remains asymptomatic with improved NYHA functional class, improved echocardiographic indices of LV function, and a further reduction in BNP. Despite postoperative CHB in the neonatal period and no demonstrable His-Purkinje automaticity thereafter, this case demonstrates that residual His-Purkinje or left bundle branch fibers may persist and be recruited later in life to achieve CSP. Furthermore, it underscores the need to refine pediatric ECG criteria for confirming LBBAP capture in complex CHD, particularly in patients with septal prosthetic material such as VSD patches.

A limitation is that the initial epicardial CRT system may have been suboptimal in terms of lead positioning. The RV lead was located on the anterosuperior RV surface and the LV epicardial lead at the apex, without the ability to achieve true lateral LV activation. This common constraint in pediatric epicardial CRT may have contributed to suboptimal resynchronization and should be considered when interpreting the degree of improvement observed after conversion to LBBAP. Larger studies with longer observation periods are needed to determine whether LBBAP may represent a viable alternative to CRT-P in postsurgical pediatric PICM.

## Conclusion

CSP, particularly LBBAP, represents a promising alternative to CRT-P for managing PICM in pediatric patients after congenital heart surgery, offering the potential to reduce ventricular dyssynchrony and enhance cardiac function. This first pediatric case of CRT to LBBAP conversion for PICM demonstrates that CSP can restore physiologic activation, reverse ventricular dysfunction, and, in selected patients, potentially surpass CRT-P in effectiveness. Despite limited long-term data, our findings highlight LBBAP as a compelling, physiology-based strategy for the management of PICM in children.

## Disclosures

The authors have no conflicts of interest to disclose.
